# *Bidens pilosa* Ethylene acetate extract can protect against L-NAME-induced hypertension on rats

**DOI:** 10.1186/s12906-017-1972-0

**Published:** 2017-10-10

**Authors:** Danielle Claude Bilanda, Paul Désiré D. Dzeufiet, Léontine Kouakep, Bibi Farouck O. Aboubakar, Léonard Tedong, Pierre Kamtchouing, Théophile Dimo

**Affiliations:** 1 Department of Animal Biology and Physiology, Laboratory of Animal Physiology, University of Yaounde I, P.O. Box 812, Yaounde, Cameroon; 2 Faculty of Health Sciences, Université des Montagnes, P.O. Box 208, Bangangté, Cameroon

**Keywords:** *Bidens pilosa*, L-name, Hypertension, Oxidative stress, Rat

## Abstract

**Background:**

Essential hypertension is mainly caused by endothelial dysfunction which results from nitric oxide (NO) deficiency. The present study was design to evaluate the protective effect of *Bidens pilosa* ethylene acetate extract (Bp) on L-NAME induced hypertension and oxidative stress in rats.

**Methods:**

Male Wistar rats were used to induce hypertension by the administration of L-NAME (a non-pecific nitric oxide inhibitor) (50 mg/kg/day). The others groups were receiving concomitantly L-NAME plus Bp extract (75 and 150 mg/kg/day) or losartan (25 mg/kg/day). All the treatments were given orally for 4 weeks. At the end of the treatment, the hemodynamic parameters were recorded using the direct cannulation method. The effects of the extract on lipid profile, kidney and liver functions as well as oxidative stress markers were evaluated by colorimetric method. Results were expressed as the mean ± SEM. The difference between the groups was compared using one-way analysis of variance (ANOVA) followed by the Duncan’s post hoc test.

**Results:**

Animals receiving L-NAME presented high blood pressure, normal heart rate and lipid profile as well as NO depletion, liver and kidney injuries and oxidative stress. The concomitant treatment with L-NAME and Bp or losartan succeeded to prevent the raised of blood pressure and all the other injuries without affecting the heart rate.

**Conclusion:**

These results confirm the antihypertensive effects of *Bidens pilosa* and highlight its protective properties in L-NAME model of hypertension in rat, probably due to the presence of Quercetin 3,3 ‘-dimethyl ether 7–0-β-D-glucopyranoside.

**Electronic supplementary material:**

The online version of this article (10.1186/s12906-017-1972-0) contains supplementary material, which is available to authorized users.

## Background

Essential (or primary) hypertension is mainly caused by endothelial dysfunction which results from nitric oxide (NO) deficiency [1]. In fact, it has been found that vascular endothelium of hypertensive patients produces less nitric oxide. NO released by endothelial cells is an important regulator of vascular function [2]. Therefore, chronic administration of Nw-nitro-L-arginine methyl ester (L-NAME) a non-pecific nitric oxide inhibitor causes arterial hypertension in rats [3]. L-NAME-induced hypertension is then a suitable model to test the vascular protective effects of antihypertensive drugs in the context of NO deficiency [3]. Plant extracts used in the treatment of many diseases contain natural antioxidants that can prevent ROS formation and its damages [4]. Moreover some flavonoid have proved to ameliorate hypertension, oxidative stress and lipid metabolism in L-NAME hypertensive rats [5].

*Bidens pilosa* which is used in the current study, is a medicinal plant belonging to the family of Asteracae with a widely occurrence in the tropical and hot areas of Africa, including Cameroon. This plant is used in traditional medicine for at least 40 categories of illnesses, including hypertension [6, 7]. The plant has already been tested for it hypotensive and antihypertensive activities on many models of hypertension such as spontaneously hypertensive rats (SHR), salt-loading and fructose-induced hypertensive rats [8–10] and even human cells [11]. The mechanism of action of the extracts from *Bidens pilosa* include vasodilatation, calcium blocker, free radicals scavenging insulin-sensibility, lipid profil improving [10–13]. The interest on bioactive compounds from plant extracts has increased in recent years due to their health benefits, particularly protection against a variety of ailments such as cardiovascular diseases [14]. *Bidens pilosa* is now knowm to contain several chemical and useful compounds, including at least 60 flavonoids [7]. In the present plant extract, we identified two bioactive flavonoids namely Quercetin 3,3 ‘-dimethyl ether 7–0-β-D-glucopyranoside and Iso-Okanin 7-O-β-D-(2 “, 4″, 6 “-triacetyl)-glucopyranoside [15]. Quercetin is known to improve vascular function in an endothelium-dependant and -independent manner [16]. Wistar rat is a suitable model of essential hypertension caused by endothelial dysfunction. Therefore, the purpose of this study was to evaluate the effects of *Bidens pilosa* ethylene acetate extract on L-NAME induced hypertension and oxidative stress in rats.

## Methods

### Animals

Male Wistar rats aged 10–12 weeks and weighing 180 to 250 g were randomly selected from our colony. They were raised in the animal house of the Faculty of Sciences, University of Yaounde I, Cameroon in Plexiglas cages. All efforts were made to minimize animal suffering and to reduce the number of animals used. Rats were housed 3 per cages and exposed to daily 12 h light – dark cycle. They were maintained in a room temperature (25 ± 3 °C) with free access to a standard animal diet and tap water. All the procedures and protocols involving animals and their care were conducted in conformity with the institutional guidelines and approved by the Cameroon National Ethical Committee (Reg. No. FWA-IRB00001954). The effects of *Bidens pilosa* ethylene acetate extract (Bp) were examined in vivo at the end of the experimentation on mean arterial blood pressure (MABP) of rats previously treated with L-NAME (See Additional file 1).

### Plant material

Fresh leaves of *Bidens pilosa* L (Asteraceae) were collected from Yaounde’s suburb (Eleveur) on September 2011 and authenticated at the National Herbarium of Cameroon by comparison to the voucher specimen (No 65112/HNC) deposited in 2005. The extraction was done as previously described [15]. Briefly, the ethyl acetate extract was prepared by macerating 1000 g of air dried leaves for two days in 5 L of methylene chloride/methanol (1:1). After filtration, the collected extract was concentrated using a rotary evaporator HEIDOLPH W2000, giving about 60 g of greenish dough. This extract was exhausted in 500 mL of ethyl acetate and after concentration using rotary evaporator, giving 12.7 g of ethyl acetate extract of *B. pilosa*. This extract was dissolved in 1% DMSO for daily used (See Additional file 1).

### L-NAME –hypertension induction and treatment

Wistar rats (30) were randomly divided into five groups of six rats. The first group (control) received a solution of DMSO (1%), given according to the weight (1 mL/200 g of weight), while the second one (L-NAME group) received L – NAME (50 mg/kg/day) plus the vehicle. The third group received at the same time L-NAME (50 mg/kg/day) and losartan (50 mg/kg/day), while the fourth and fifth groups received a combination of L-NAME (50 mg/kg/day) and *Bidens pilosa* ethylene acetate extract (Bp: 75 and 150 mg/kg/day respectively). All the treatments were administered daily orally for 4 weeks at the corresponding volume of 1 mL/200 g, from 8 to 8.30 AM using oesophageous cannula (See Additional file 1).

### Hemodynamic parameters recording

At the end of the respective treatment, arterial blood pressure and heart rate of all rats were measured as previously described [17]. Briefly, the rat was anesthetized using an intraperitoneal injection of urethane (1.5 g/kg). The trachea was exposed and cannulated to facilitate spontaneous breathing. The arterial blood pressure was measure from carotid artery via an arterial cannula connected to a pressure transducer coupled with a hemodynamic recorder Biopac Student Lab. (MP35) and computer (See Additional file 1).

### Blood and organs collection

Immediately after hemodynamic parameters measurement, blood samples were collected from the abdominal artery, and centrifuged at 3000 rpm for 15 min. The plasma obtained was kept at −20 °C for biochemical analysis. Thereafter, the heart, the kidney, the liver and the thoracic aorta were collected, washed in saline and weighed and kept for oxidative stress markers and NO evaluation.

### Biochemical analysis

Heart, aorta, liver and kidney were dissected out and homogenized in Mc Even solution for heart and aorta or in Tris–HCl 50 mM buffer solution for liver and kidney (20%, *w*/*v*). Tissue levels of reduced glutathione (GSH), superoxide dismutase activity (SOD) and malondylaldehyde (MDA) were assayed using colorimetric method as described by Ellman, [18], Misra and Fridovich [19] and Wilbur et al. [20] respectively. The tissue concentration of nitric oxide (NO) was evaluated using the Griess method [21]. The concentrations of total cholesterol (TC), high density lipoprotein (HDL) cholesterol and triglycerides (TG), creatinin and bilirubine levels in serum were determined using commercial diagnostic kits (Fortress, UK). Atherogenic index was calculated following the formula used by Wakayashi and Kobaba [22]. The activities of alanine and aspartate aminotransaminases were also determined spectrophotometrically using commercial diagnostic kits (Fortress, UK).

### Statistical analysis

Results were expressed as the mean ± SEM. The difference between the groups was compared using one-way analysis of variance (ANOVA) followed by the Duncan’s post hoc test. A value of *p* < 0.05 was considered statistically significant.

## Results

### Effects of ethylene acetate extract of *Bidens pilosa* on arterial blood pressure and heart rate

Animals that received only L-NAME (50 mg/kg/day) were hypertensive after four weeks of treatment. Concomitant administration of L-NAME with losartan (Los + L-NAME) or *Bidens pilosa* (Bp 75 + L-NAME and Bp 150 + L-NAME) dose-dependently and significantly prevented the installation of hypertension as depicted in Table 1. The mean arterial blood pressure (MABP) value was 166.18 ± 2.68 mmHg in L-NAME treated rats versus 128.43 ± 1.45 mmHg, 126.69 ± 8.12 and 110.03 ± 8.19 mmHg in the Los + L-NAME, Bp 75 + L-NAME and Bp 150 + L-NAME groups respectively. However, no significant change in heart rate was observed between experimental groups (Table 1).

**Table 1:** Effects of ethylene acetate extract of *Bidens pilosa* on blood pressure and heart rate

Parameters	Treatments				
	Vehicle (10 ml/kg)	L-NAME	L-NAME + Bp (75 mg/kg)	L-NAME + Bp (150 mg/kg)	L-NAME + Losartan
DBP (mm Hg)	104.14 ± 7.42	158.96 ± 2.29^b^	122.22 ± 8.04^β^	105.28 ± 8.22^β^	123.79 ± 1.32^β^
SBP (mm Hg)	117.56 ± 8.66	180.62 ± 4.08^b^	135.64 ± 8.56^β^	119.52 ± 8.20^β^	137.72 ± 3.20^β^
MABP (mmHg)	108.61 ± 7.75	166.18 ± 2.68^b^	126.69 ± 8.12^β^	110.03 ± 8.19^β^	128.43 ± 1.45^β^
HR (beat /min)	334.21 ± 10.51	311.48 ± 14.14	314.02 ± 13.14	352.20 ± 15.74	345.30 ± 12.82

Each value represents a means ± S.E.M. of 6 rats; ^b^ *P* < 0.01, significantly different compared to normal rats. ^β^ *P* < 0.01, significantly different compared to L-NAME hypertensive rats

*DBP* diastolic blood pressure, *SBP* systolic blood pressure, *MABP* mean arterial blood pressure, *HR*heart rate

### Effects of ethylene acetate extract of *Bidens pilosa* on lipid profile

The oral administration of L-NAME (50 mg/kg) to rats resulted in an elevated plasma lipid profile (Table 2). L-NAME treated rats showed significantly (*p* < 0.01) higher levels of plasma triglycerides, LDL-cholesterol atherogenic index and significantly (*p* < 0.05) lower levels of HDL- cholesterol as compared to the normal control rats. The *Bidens pilosa* ethylene acetate extract (Bp 75 and 150 mg/kg/day) significantly modulated the lipid profile in animals receiving concomitantly L-NAME. Bp extract reduced plasma triglycerides and LDL- cholesterol by 51.29%, 78.57% and 46.42%, 48.21% respectively as compared to L-NAME hypertensive rats. Bp also prevented the raise in atherogenic index. The extract increased the level of plasma HDL-cholesterol by 25.75% and 9.09% with the doses 75 and 150 mg/kg respectively as compared to L-NAME hypertensive rats. In the same conditions, losartan prevented the decrease of HDL-cholesterol by 18.18% (*p* < 0.05), and the increase of LDL-cholesterol by 28.77% (p < 0.05) and triglycerides by 58.44% (*p* < 0.01) as compared to L-NAME hypertensive rats.

**Table 2:** Effects of ethylene acetate extract of *Bidens pilosa* on lipid profile

Parameters	Treatments				
	Vehicle (10 ml/kg)	L-NAME	L-NAME + Bp (75 mg/kg)	L-NAME +Bp (150 mg/kg)	L-NAME + Los (25 mg/kg)
Total Chol (mmol/L)	1.14 ± 0.10	1.22 ± 0.07	1.13 ± 0.12	1.00 ± 0.14	1.18 ± 0.05
HDL chol (mmol/L)	0.91 ± 0.13	0.66 ± 0.10^a^	0.83 ± 0.11^α^	0.72 ± 0.16	0.78 ± 0.09
LDL chol (mmol/L)	0.24 ± 0.06	0.56 ± 0.08^a^	0.30 ± 0.04	0.29 ± 0.03^β^	0.40 ± 0.12
Triglycerides (mmol/L)	0.61 ± 0.06	1.54 ± 0.05^b^	0.75 ± 0.27^α^	0.33 ± 0.11^β^	0.64 ± 0.22^β^
Atherogenic index (mmol/L)	0.34 ± 0.01	0.67 ± 0.07^a^	0.39 ± 0.08^α^	0.28 ± 0.05^β^	0.51 ± 0.07

Each value represents a means ± S.E.M. of 6 rats; ^a^ *P* < 0.05 ^b^P < 0.01, significantly different compared to normal rats. ^α^ *P* < 0.05, ^β^P < 0.01, significantly different compared to L-NAME hypertensive rats

### Effects of ethylene acetate extract of *Bidens pilosa* on liver and kidney functions

As shown in Table 3 The oral administration of L-NAME (50 mg/kg) to rats resulted in an increase of plasma AST and ALT by 106.92% and 58.34% (p < 0.01) respectively. In L-NAME hypertensive rats it is also observed an increase, but not significant in creatinin (10%) and bilirubine (35.32%) as well as a slight drop in proteins (13.53%) as compared to nornotensive rats group. The combine administration of L-NAME and Bp (75 mg/kg et 150 mg/kg) prevented that increase for AST by 82.69% and 30.27% and for ALT by 70.14% and 80.38% respectively as compared to L-NAME hypertensive rats. Losartan induced a significant decrease of the plasma level of AST by 80.75% and of AST by 38.79% as compared to L-NAME hypertensive group. Bp extract prevented the increase induced by L-NAME of plasma creatinin by 35.22% at the dose of 75 mg/kg and by 28.40% at the dose of 150 mg/kg as compared to hypertensive group.

**Table 3:** Effects of BPEA on liver and kidney functions

Parameters	Treatments				
	Vehicle (10 ml/kg)	L-NAME (50 mg/kg)	L-NAME + Bp (75 mg/kg)	L-NAME + Bp (150 mg/kg)	L-NAME + Los (25 mg/kg)
AST (UI)	31.03 ± 2.08	64.21 ± 6.73^b^	11.11 ± 0.87^bβ^	45.68 ± 5.16^aβ^	12.36 ± 1.07^aβ^
ALT (UI)	18.51 ± 1.78	29.31 ± 2.30^b^	11.75 ± 1.15^bβ^	10.72 ± 0.21^bβ^	17.94 ± 2.86^β^
Creatinin (mg/mL)	0.80 ± 0.06	0.88 ± 0.68	0.57 ± 0.06	0.63 ± 0.18	0.87 ± 0.45
Bilirubine (mg/L)	4.02 ± 1.38	5.44 ± 1.707	5.63 ± 0.92	5.71 ± 1.058	8.04 ± 1.79^α^
Proteins (mg/mL)	81.84 ± 2.57	70.76 ± 14.69	75.04 ± 14.10	85.20 ± 12.79	71.32 ± 15.94

Each value represents a means ± S.E.M. of 6 rats; ^a^P < 0.05 ^b^P < 0.01, significantly different compared to normal rats. ^α^P < 0.05, ^β^P < 0.01, significantly different compared to L-NAME hypertensive rats

### Effects of ethylene acetate extract of *Bidens pilosa* on oxidative stress parameters

The level of malondialdehyde (MDA) significantly (*p* < 0.01) increased in aorta, heart, liver and kidneys of rats which received L-NAME compared to control normotensive group. Apart from aorta and liver with Bp (75 mg/kg), the combined treatment with Bp (75 and 150 mg/kg/day) for 4 weeks significantly prevented the rise in tissue MDA levels in the rest of investigated organs as compared to L-NAME hypertensive rats. The same effect was observed with losartan (25 mg/kg/day).

Treatment of rats with L-NAME dropped significantly the glutathione (GSH) level in aorta, kidneys, liver, (*p* < 0.001) and heart (*p* < 0.05) when compared with control group. So, the decline percentages were 76.92, 34.13, 57.65 and 54.873% respectively in aorta, heart liver and kidney. The L-NAME treatment associated with Bp extract or losartan has significantly prevented that decrease in all the investigated organs, except with the lower dose of Bp (75 mg/kg) in the heart, the liver and the kidney. The same Effect has been observed with losartan in the kidney when compared with L-NAME hypertensive rats.

The administration of L-NAME in rats significantly decreased activity of SOD in aorta, heart, liver (*p* < 0.01) and kidneys (p < 0.05), when compared to control group. The values of decline percentages were 38.80, 52.08, 54.36 and 33.54% in aorta, heart liver and kidney respectively as compare to control normotensive rats group. Bp extract at the doses of 75 and 150 mg/kg blunted significantly the decrease in SOD activity in heart and liver (p < 0.01) observed in hypertensive group. Losartan also blunted significantly (p < 0.01) the decrease of SOD in heart and liver.

**Fig. 1:**
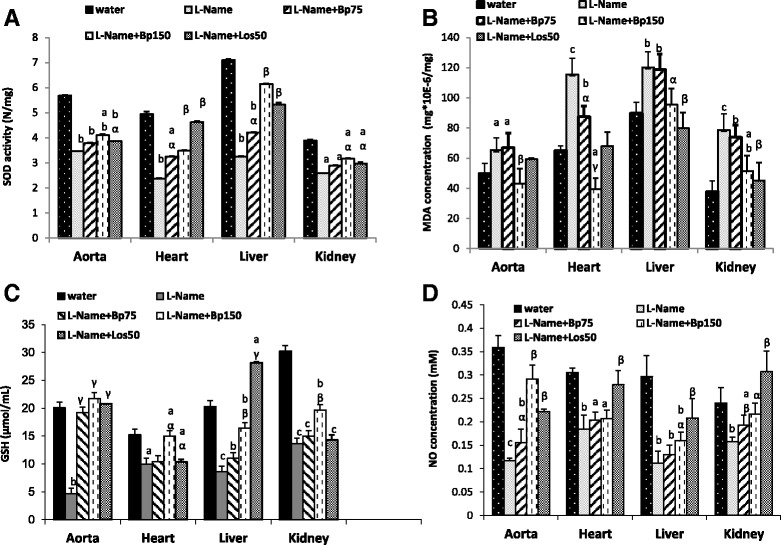
Effect of *Bidens pilosa* on oxidative stress parameters and NO of L-NAME-induced hypertensive rats; SOD (**a**), MDA (**b**), GSH (**c**) and NO (**d**). Each bar represents a means ± S.E.M. of 6 rats; ^a^ *p* < 0.05, ^b^ *p* < 0.01, ^c^ *p* < 0.001, significantly different compared to normal rats. ^α^p < 0.05, ^β^p < 0.01, ^γ^p < 0.001, significantly different compared to L-NAME hypertensive rats

Aortic, cardiac, liver and kidney content in NO are represented in Fig. 1d. In rats treated only with L-NAME, all the NO concentration decrease significantly (p < 0.01) by 38.16, 38.84, 62.28 and 34.16% in aorta heart liver and kidney respectively as compared to the control normotensive rats. The Bp extract dose-dependently prevented the deleterious effects of L-NAME in the tissue NO content. It prevented by 33.33, 10.86, 16.07 and 22.15% at the dose 75 mg/kg/day and by 148.71, 12.5, 42.85 and 36.70% at the dose 150 mg/kg/day the concentration of NO in the aortic, cardiac liver and kidney tissues respectively as compared to the L-NAME hypertensive group. Losartan had a similar effect.

## Discussion

This study investigated the antihypertensive effect of *Bidens pilosa* ethylene acetate (Bp) extract in L-NAME-induced hypertensive rats and its effects on oxidative stress as well as its damages on liver and kidney. The sustained L-NAME induced hypertension was confirmed [23]. The fact that Bp could prevent hypertension induced by L-NAME indicates that it may act directly or no in NO production or activity. As already mentioned, *Bidens pilosa* extract used in the present study contains many flavonoids namely Quercetin 3,3 ‘-dimethyl ether 7–0-β-D-glucopyranoside and Iso-Okanin 7-O-β-D-(2 “, 4″, 6 “-triacetyl)-glucopyranoside. Previous studies have demonstrated that quercitine was able to improve endothelium function by increasing the bioavaibility and/or the production of NO [16]. In addition quecitine isolated from *Bidens pilosa* can prevent and attenuate hypertension [24]. This ability passes trough its vasodilatator, cardio protective and vasoprotective effects as well as its antioxidant properties [10, 11, 13]. Quecitine can also prevent cell smooth muscle proliferation and migration [25]. We can also think of the calcium blocker activity of the plant that has been demonstrated [26]. All this might have contributed at least in part in the process of the plant extract to prevent the induction of hypertension by L-NAME administration. The fact that none of the treatment has affected the heart rate is in accordance with previous studies that have shown that, the chronic administration of L -NAME did not alter the heart rate [27]. Since no changes in the heart rate were also found in rats treated concomitantly with L-NAME and Bp extract, it seems that the lowering of blood pressure may be predominantly due to its effects on vessels. Though Dimo et al. [13] have demonstrated that *Bidens pilosa* reduced heart rate and the force of contraction; other studies using other extracts of *Bidens pilosa* showed that the plant does not really affect the heart, but mostly the vessels [8]. This difference may be due to the difference in extraction. Therefore, we can suggest that the antihypertensive effect of our plant extract might be mainly due to its ability to reduce the peripheral resistance via its vasodilating activities [8].

The fact that the dyslipidemia observed in L-NAME-induced hypertensive rats, was significantly managed by both losartan and BPEA confirmed both the hypotriglyceridemic and hypocholesterolemic properties of the plant extract [9, 10, 12]. In fact, L-NAME elevates the serum concentrations of free fatty acid by lowering the activity of hepatic carnitine palmitoyltransferase, the rate-limiting enzyme of fatty acid oxidation, leading to hyperlipidemia [28]. The elevation in serum triglyceride and cholesterol might be due to reduced fatty acid oxidation [28]. The hypolidemic potential of *Bidens pilosa* may therefore be due, at least in part, to the stimulation of fatty acid oxidation, related to the presence of quercetin which may be involved in lipid metabolism [29]. Quercitine is also known to reduce triglyceride circulation level [30]. This in turn might contribute to the cardiovascular protective effects of Bp. The antilipidemic properties of Bp extract could also be due to its antioxidant composition [7]. In effect, in our study, the oxidative status was impaired in L-NAME treated group. The association of Bp or losartan with L-NAME prevented the decrease of glutathione and SOD and the increase of MDA in treated rats. Glutathione plays an excellent role in protecting cell from oxidative damages. Therefore Bp may be acting as membranes protector to prevent the lipid peroxidation or as free radicals scavenger [16]. That action of our plant extract may in turn protect the organs against the injuries caused by free radicals. The antioxidant potential of Bp might also be due to the free radical scavenging properties of its known phytochemicals such as flavonoids, phenylpropanoids and phenols [6, 7]. This can then explain the protective effect of our extract on liver and kidney. In the present study, we evaluated the toxicity L-NAME-induced hypertension on these two organs by assessing the level of blood creatinine, bilirubine and transaminases (AST and ALT). *Bidens pilosa* inhibited the elevation in serum levels of AST, ALT and bilirubine induced by L-NAME; that is similar with the effect on oxidative stress markers. This may indicate that oxidative stress contributes in the mechanism(s) of hepatotoxicity due to chronic consumption of L-NAME. The low level of serum enzyme activity following the concomitant treatment with Bp as compared to untreated rats confirms the liver and kidney protective effects of *Bidens pilosa* [6] as well as its antioxidant activity [7]. In the present study, treatment with L-NAME increased the blood pressure in association with decreased in heart and aortic NO tissue levels. The levels of heart and aortic NO were reversed by co-administration of Bp showing the protective effect of the plant extract on the vascular endothelium. This beneficial effect of the plant extract may be associated with the presence of substances which are reported to improve vascular function by endothelium-dependent and independent manner and increase NO bioavailability and/or production [16, 31].

## Conclusion

These results showed that the ethylene acetate extract of *Bidens pilosa* leaves might prevent the L-NAME-induced hypertension in rats. Current findings also confirm that *Bidens pilosa* is able to normalize lipid profile, fight against oxidative stress and cell damage in liver and kidney and to improve endothelial function in this animal model of hypertension. These results are probably due to the presence of Quercetin 3,3′-dimethyl ether 7–0-β-D-glucopyranoside and scientifically confirm the antihypertensive and antioxidative properties of the aqueous extract of *B. pilosa* as used in Cameroonian traditional medicine.

## Additional file

Additional file 1: Animal handling procedure. (DOCX 615 kb)

## Abbreviations

ALT: Alanine amino-transferase
AST: Aspartate amino-transferase
Bp: *Bidens pilosa* ethylene acetate extract
DBP: Diastolic blood pressure
GSH: Reduced glutathione
HDL-c: High density lipoprotein cholesterol
HR: Heart rate
LDL-c: Low density lipoproteins cholesterol
L-NAME: Nw-nitro-L-arginine methyl ester
MABP: Mean arterial blood pressure
MDA: Malondialdehyde
NO: Nitric oxide
SBP: Systolic blood pressure
SOD: Superoxide dismutase activity
