# Supplementary material for: Bidens pilosa Ethylene acetate extract can protect against L-NAME-induced hypertension on rats
Source: BMC Complement Altern Med. 2017 Oct 10;17:479. doi: 10.1186/s12906-017-1972-0 (PMC5633871; doi:10.1186/s12906-017-1972-0)
Supplement: Additional file 1: — Animal handling procedure. (DOCX 615 kb) [file 12906_2017_1972_MOESM1_ESM.docx]

Additional file 1

**Methods**

**I- Animals**

Male Wistar rats aged 10 – 12 weeks and weighing 180 to 250 g were randomly selected from our colony. They were raised in the animal house of the Faculty of Sciences, University of Yaounde I, Cameroon in Plexiglas cages. All efforts were made to minimize animal suffering and to reduce the number of animals used. Rats were housed 3 per cages and exposed to daily 12 hours light – dark cycle. They were maintained in a room temperature (25 ±3 °C) with free access to a standard animal diet and tap water. All the procedures and protocols involving animals and their care were conducted in conformity with the institutional guidelines and approved by the Cameroon National Ethical Committee (Reg. No. FWA-IRB00001954). The effects of *Bidens pilosa* ethylene acetate extract (BPEA) were examined *in vivo* at the end of the experimentation on mean arterial blood pressure (MABP) of rats previously treated with L-NAME.

**II- Plant material**

Fresh leaves of *Bidens pilosa* L (Asteraceae) were collected from Yaounde's suburb (Eleveur) on September 2011 and authenticated at the National Herbarium of Cameroon by comparison to the voucher specimen (No 65112/HNC) deposited in 2005. The extraction was done as previously described [9]. Briefly, the ethyl acetate extract was prepared by macerating 1000 g of air dried leaves for two days in 5 L of methylene chloride/methanol (1:1). After filtration, the collected extract was concentrated using a rotary evaporator HEIDOLPH W2000, giving about 60 g of greenish dough. This extract was exhausted in 500 mL of ethyl acetate and after concentration using rotary evaporator, giving 12.7 g of ethyl acetate extract of *B. Pilosa*. This extract was dissolved in 1 % DMSO for daily used.

**III- L-NAME –hypertension induction and treatment**

Wistar rats (30) were randomly divided into five groups of six rats. The first group (control) received a solution of DMSO (1 %), given according to the weight (1 mL/200 g of weight), while the second one (L-NAME group) received L – NAME (50 mg/kg/day) plus the vehicle. The third group received at the same time L-NAME (50 mg/kg/day) and losartan (25 mg/kg/day), while the fourth and fifth groups received a combination of L-NAME (50 mg/kg/day) and *Bidens pilosa* ethylene acetate extract (BPEA: 75 and 150 mg/kg/day respectively). All the treatments were administered daily orally for 4 weeks at the corresponding volume of 1 mL/200 g, from 8 to 8.30 AM using oesophageous cannula.

**IV- Hemodynamic parameters recording**

At the end of the respective treatment, arterial blood pressure and heart rate of all rats were measured as previously described [11]. Brieﬂy, the rat was anesthetized using an intraperitoneal injection of urethane (1.5 g/kg; 1 mL/100 g of body weight). The trachea was exposed and cannulated to facilitate spontaneous breathing. The arterial blood pressure was measure from carotid artery *via* an arterial cannula connected to a pressure transducer coupled with a hemodynamic recorder Biopac Student Lab. (MP35) and computer. Urethane is appropriate for this experiment because it does not depress the respiratory function.

**Pictures**


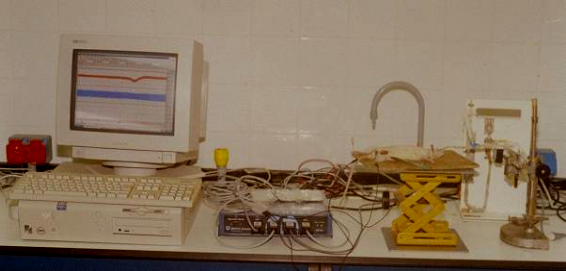


**Hemodynamic parameters recording**

**V- Blood and organs collection**

Immediately after hemodynamic parameters measurement, blood samples were collected from the abdominal artery in sterile glass tubes, and centrifuged at 3000 rpm for 15 minutes. The plasma obtained was kept at −20°C for biochemical analysis. Thereafter, the heart, the kidney, the liver and the thoracic aorta were collected, washed in saline and weighed and kept for oxidative stress markers and NO evaluation.

**VI- Biochemical analysis**

Heart, aorta, liver and kidney were dissected out and homogenized in Mc Even solution for heart and aorta or in Tris–HCl 50 mM buffer solution for liver and kidney (20%, w/v). Tissue levels of reduced glutathione (GSH), superoxide dismutase activity (SOD) and malondylaldehyde (MDA) were assayed using colorimetric method as described by Ellman, [12], Misra and Fridovich [13] and Wilbur *et al*. [14] respectively. The tissue concentration of nitric oxide (NO) was evaluated using the Griess method [15]. The concentrations of total cholesterol (TC), high density lipoprotein (HDL) cholesterol and triglycerides (TG), urea, creatinin and bilirubine levels in serum were determined using commercial diagnostic kits (Fortress, UK). Atherogenic index was calculated following the formula used by Wakayashi and Kobaba [16]. The activities of alanine and aspartate aminotransaminases were also determined spectrophotometrically using commercial diagnostic kits (Fortress, UK).

Results were expressed as the mean ± SEM. The difference between the groups was compared using one-way analysis of variance (ANOVA) followed by the Duncan’s post hoc test. A value of p < 0.05 was considered statistically significant.
